# Formulation, characterization, and cellular toxicity assessment of tamoxifen-loaded silk fibroin nanoparticles in breast cancer

**DOI:** 10.1080/10717544.2021.1958106

**Published:** 2021-07-30

**Authors:** Afrasim Moin, Shahid Ud Din Wani, Riyaz Ali Osmani, Amr S. Abu Lila, El-Sayed Khafagy, Hany H. Arab, Hosahalli V. Gangadharappa, Ahmed N. Allam

**Affiliations:** a Department of Pharmaceutics, College of Pharmacy, University of Hail, Hail, Saudi Arabia; b Department of Pharmaceutics, JSS College of Pharmacy, JSS Academy of Higher Education and Research, Mysuru, India; c Department of Pharmaceutics, CT Institute of Pharmaceutical Sciences, Jalandhar, India; d Department of Biosciences and Bioengineering, Indian Institute of Technology Bombay, Mumbai, India; e Department of Pharmaceutics, Faculty of Pharmacy, Zagazig University, Zagazig, Egypt; f Department of Pharmaceutics, College of Pharmacy, Prince Sattam Bin Abdulaziz University, Al-kharj, Saudi Arabia; g Department of Pharmaceutics and Industrial Pharmacy, Faculty of Pharmacy, Suez Canal University, Ismailia, Egypt; h Department of Pharmacology and Toxicology, College of Pharmacy, Taif University, Taif, Saudi Arabia; i Department of Pharmaceutics, Faculty of Pharmacy, Alexandria University, Alexandria, Egypt

**Keywords:** Breast cancer, *in vitro* cytotoxicity, nanoparticles, silk fibroin, tamoxifen citrate

## Abstract

Silk fibroin (SF) is a natural polymeric biomaterial that is widely adopted for the preparation of drug delivery systems. Herein, we aimed to fabricate and characterize SF nanoparticles loaded with the selective estrogen receptor modulator; tamoxifen citrate (TC-SF-NPs) and to assess their *in vitro* efficacy against breast cancer cell lines (MCF-7 and MDA-MB-231). TC-loaded SF-NPs were characterized for particle size, morphology, entrapment efficiency, and release profile. In addition, we examined the *in vitro* cytotoxicity of TC-SF-NPs against human breast cancer cell lines and evaluated the anticancer potential of TC-SF-NPs through apoptosis assay and cell cycle analysis. Drug-loaded SF-NPs showed an average particle size of 186.1 ± 5.9 nm and entrapment efficiency of 79.08%. Scanning electron microscopy (SEM) showed the nanoparticles had a spherical morphology with smooth surface. Tamoxifen release from SF-NPs exhibited a biphasic release profile with an initial burst release within the first 6 h and sustained release for 48 h. TC-SF-NPs exerted a dose-dependent cytotoxic effect against breast cancer cell lines. In addition, flow cytometry analysis revealed that cells accumulate in G_0_/G_1_ phase, with a concomitant reduction of S- and G_2_-M-phase cells upon treatment with TC-SF-NPs. Consequently, the potent anticancer activities of TC-SF-NPs against breast cancer cells were mainly attributed to the induction of apoptosis and cell cycle arrest. Our results indicate that SF nanoparticles may represent an attractive nontoxic nanocarrier for the delivery of anticancer drugs.

## 1. Introduction

Breast cancer represents one of the significant ongoing public health issues and is the most familiar non-cutaneous malignancy among women in US (Grobmyer et al., 2012). It is the second leading cancer after lung cancer worldwide. About 20% of the cancer patients suffer from breast cancer and it is the main death cause for women (Riihimäki et al., 2012). The American Cancer Society (ACS) reports the number of new cancer cases and deaths annually and compiles the most recent statistics on cancer prevalence, survival, and mortality in the US. According to ACS, about one out of eight women will be identified with invasive breast cancer during their lifetime and one out of 39 women will die from breast cancer.

**Figure 1.**
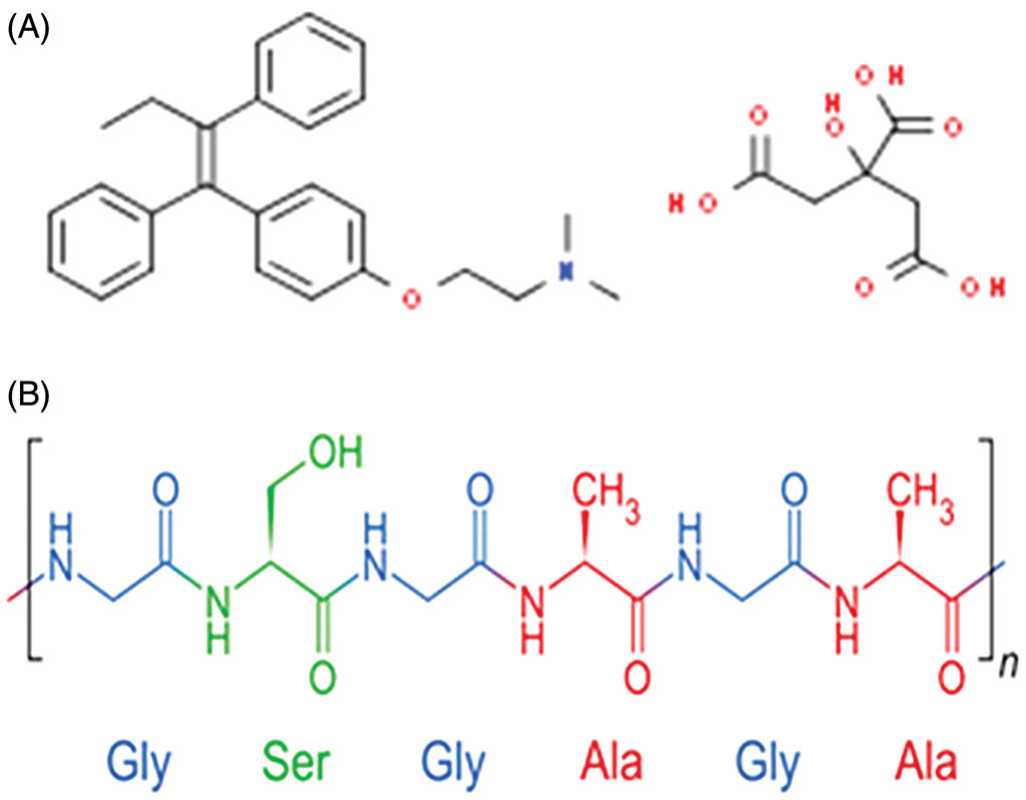
Chemical structure of (A) tamoxifen citrate and (B) silk fibroin.

Tamoxifen is a potent hydrophobic endocrine drug commonly used to treat breast cancers and patients at high risk (Day et al., 2020; Abbasalipourkabir et al., 2016). Tamoxifen can act as estrogenic or antiestrogenic, depending on the dosage and the tissues targeted (Figure 1(A)). Nevertheless, tamoxifen therapeutic efficacy is adversely compromised by its poor oral bioavailability, presumably due to extensive hepatic metabolism (Jordan, 2007). In addition, long-term tamoxifen administration can cause adverse side effects, including the development of endometrial cancer, hepatic cancer, increased blood clotting, ocular retinopathy, and corneal opacity (Parkkari et al., 2003; Layek & Mukherjee, 2010). These undesirable side effects of tamoxifen, along with numerous obstacles to the successful delivery of the drug to the tumor site, require the development of a targeted delivery system that grants site-specific delivery of the drug while minimizing the nonspecific off-target effects. Accordingly, an array of nanoparticulate delivery systems have been explored for their efficacy to deliver tamoxifen to the tumor cells, such as nanospheres and nanoparticles comprising poly-caprolactone (Łukasiewicz et al., 2021; Chawla & Amiji, 2002; Villemson et al., 2006). The motive behind the use of such biocompatible and biodegradable polymeric nanocarriers is to augment the selective delivery of drug dose to tumor site while sparing healthy tissues from drug’s off-target effect.

Recently, polymeric drug delivery systems have gained increased attention as an effective alternative to conventional formulations. They offer a mean of providing a reservoir to the active pharmaceutical ingredients, improving their physicochemical properties, and circumventing some of the major obstacles faced in drug delivery, such as biocompatibility, intracellular delivery, and targeting specificity in order to ensure the therapeutic efficacy and improving patients’ quality of life (Al Saqr et al., 2021a,b; Vishwa et al., 2021; Luo et al., 2019). An ideal drug delivery system should stabilize the entrapped drug, offer tunable release kinetics, and mitigate the undesirable side effects *via* ensuring site-specific targeting, particularly in the case of highly toxic drugs, such as anticancer drugs.

Silk fibroin (SF), from the *Bombyx mori* silkworm, is a natural polymeric biomaterial with distinctive mechanical and physicochemical properties, rendering it a promising biomaterial for pharmaceutical and biomedical applications (Dyakonov et al., 2012; Pham & Tiyaboonchai, 2020). It has been extensively used for preparing new-fangled drug delivery systems owing to its amphiphilic chemistry, biodegradability, biocompatibility, and tunable drug release properties (Mottaghitalab et al., 2015; Wani & Veerabhadrappa, 2018). Several types of SF-based drug delivery systems have been introduced in the pharmaceutical field including; films, hydrogels, lyophilized sponges, nanofibers as well as micro and nanoparticles (Qi et al., 2017; Wang et al., 2010; Terada et al., 2016; Chirila et al., 2017). Among them, SF nanoparticles have been studied the most, particularly for anticancer drugs (Montalbán et al., 2018; Tian et al., 2014; Li et al., 2020). Generally, nanoparticles based systems are well-recognized to improve drug accumulation within tumor tissue *via* the enhanced permeation and retention (EPR) effect (Greish, 2010). In addition, nanoparticles can be designed into smart systems, entrapping therapeutic and imaging agents along with bearing stealth property (Osmani et al., 2017). Furthermore, they can selectively deliver drugs to target tissues in a controlled manner (Vishwa et al., 2021; Huang et al., 2020). SF nanoparticles showed the potential for entrapping both hydrophilic and hydrophobic drug along with controlling the release of the entrapped drug at the site of action. The hydrophobic amino acid residues in SF molecules, such as tyrosine, glycine, and alanine (Figure 1(B)) efficiently permit the entrapment of hydrophobic drugs *via* hydrophobic interaction and π–π stacking. Whilst, the hydrophilic amino acid residues in SF molecules, such as aspartic acid, serine, and glutamate, enhance their water solubility and permit the formation of nanoparticles in aqueous solutions (Li et al., 2016; Roh et al., 2019). Recently, many reports have emphasized the employment of SF-based nanoparticles for the delivery of various types of drugs, such as doxorubicin (Seib et al., 2013; Xiao et al., 2016), paclitaxel (Wu et al., 2013), cisplatin (Lozano-Pérez et al., 2015), and curcumin (Montalbán et al., 2018) as well as pDNA (Liu et al., 2017) to different types of cells. SF nanoparticles offered the advantages of being biocompatible, biodegradable along with mitigating side effects, extending blood circulation time, and controlling drug release in a site-specific and/or time-specific manner.

In this study, we developed SF nanoparticles as a biocompatible and degradable nanocarrier for loading the selective estrogen receptor modulator; tamoxifen citrate (TC). The prepared TC-loaded silk fibroin nanoparticles (TC-SF-NPs) were characterized and were assessed for its stability and *in vitro* release profile. In addition, *in vitro* cytotoxicity/apoptosis studies of TC-SF-NPs against breast cancer cell lines were assessed.

## 2. Materials and methods

### 2.1. Materials

Silk cocoons were procured from Central Sericultural Research Institute (Mysuru, India). TC was a generous gift sample from Bio Xera Private Limited (Mumbai, India). Dialysis cassette (Slyde-A-Lyzer, 3.5 kDa MWCO, ThermoFisher, Waltham, MA). Lithium Bromide was purchased from Axiom Chemicals (Gujarat, India). All other reagents were of analytical grade.

### 2.2. Silk fibroin isolation, purification, and determination of molecular mass

Aqueous SF solution was prepared as previously described with slight modifications (Zhao et al., 2015). Briefly, cocoons were sliced into small pieces and were degummed twice in boiled 0.02 M Na_2_CO_3_ solution for 30 min, followed by extensive washing with deionized water. The degummed SF was dissolved in 9.3 M lithium bromide solution at 60 °C for 5 h. The resultant solution was dialyzed against deionized water for 72 h using dialysis cassettes (MWCO 3.5 kDa) to remove salts. Subsequently, the aqueous SF solution was centrifuged at 10000 rpm 15 min and was stored at 2–8 °C. The final concentration of SF was determined by weighing the solid SF obtained after drying at 60 °C for 12 h. Dried SF was diluted with sterilized H_2_O prior to use.

### 2.3. Preparation of tamoxifen citrate-loaded silk fibroin nanoparticles

TC-SF-NPs were prepared by using desolvation method (Kundu et al., 2010). Briefly, 10 mg TC was initially dissolved in 5 ml DMSO. The resulting solution was added drop-wise to 5 ml of aqueous SF solution (2 mg/ml) under continuous stirring at 5000 rpm. After desolvation, the spontaneously formed TC-SF-NPs were centrifuged twice at 12,000 rpm for 15 min to prevent aggregation. Further, TC-SF-NPs were purified by washing trice with deionized H_2_O. The purified TC-SF-NPs were re-dispersed in deionized H_2_O and subjected to sonication (30% amplitude, 15 min) to get the desired size. The final product was finally lyophilized at 1 × 10^−4 ^ Torr and −55 °C and the resultant lyophilized NPs were stored at 2–8 °C for further experiments. Ten batches of nanoparticle formulations were prepared by varying the drug to polymer ratio.

To visualize SF-NPs cellular uptake and flow cytometry studies, the fluorescent dye propidium iodide (PI) was used to label SF-NPs. 100 µl of aqueous PI solution (1 mg/m1) was added dropwise into the SF-NPs dispersion, and stirred for 24 h at 4 °C. The unbound PI was removed from the mixture by centrifugation at 5000 rpm for 30 min, and the solid residue was re-dispersed in deionized water. All formulations were freshly prepared just before use.

### 2.4. Characterization of TC-SF-NPs

#### 2.4.1. Determination of particle size and zeta potential

Particle size and zeta potential of the prepared TC-SF-NPs were determined in triplicate using Malvern Zetasizer (Neon Series –ZS90, UK) at an angle of 90° at 25 °C (Al Saqr et al., 2021a,b).

#### 2.4.2. Morphology study

The surface morphology of TC-SF-NPs was visualized using both scanning electron microscopy (SEM) and high-resolution transmission electron microscopy (TEM). For SEM analysis, dried samples were positioned on SEM crust dock and were coated with gold in an ion-sputter. High-resolution images of TC-SF-NPs were obtained by scanning of the dock at different magnifications using HITACHI S-3400N SEM equipped with the NORAN System version 7 analytical software (Tokyo, Japan) (Seib et al., 2013). For TEM study, a drop of aqueous solution of TC-DF-NPs was placed on carbon smeared racks and kept to dry at room temperature prior to visualization under HR-TEM operating at 220 kV (Tian et al., 2014).

#### 2.4.3. Determination of drug entrapment efficiency and drug loading

Of 10 mg of developed TC-SF-NPs were dissolved in 10 ml of calcium chloride:ethanol:water ternary solvent mixture at 1:2:8 molar ratio respectively (at a temperature of 55 °C for around 1 h) and allowed to attain equilibrium solubility. The drug solution was filtered and drug concentration in the filtrate was quantified by high-performance liquid chromatography (HPLC) system. The drug loading percentage and the entrapment efficiency were calculated using the following equations:

$$\text{\%Drug loading}=(\frac{\text{Mass of drug in NPs}}{\text{Mass of NPs recovered}})\times 100$$

$$\text{Entrapment Efficiency }(\text{EE \%})=\frac{\text{ Weight of drug in NPs}}{\text{Weight of total drug added}}\times 100$$

#### 2.4.4. HPLC method for tamoxifen citrate determination

HPLC system (Shimadzu, Kyoto, Japan) equipped with a Kromasil C18 column was utilized to estimate TC concentration. The mobile phase composed of phosphate buffer and acetonitrile (55:45 v/v; pH 4.4). The detection was conducted using UV–Vis detector set at a wavelength of 280 nm. TC concentration was calculated from a pre-constructed calibration curve of TC at different concentrations.

#### 2.4.5. Infrared spectra and X-ray diffraction

Infrared spectra of SF, pure TC, physical mixture (TC and SF), and TC-SF-NPs were recorded with FT-IR spectroscopy (Shimadzu-8400, Tokyo, Japan) using the KBr disk pellet technique. The samples were scanned at a resolution of 1 cm^−1^ and scanning region of 400–4000 cm^−1^ (Bhosale et al., 2021).

X-ray diffraction (XRD) patterns of SF, pure TC, physical mixture (SF and TC), and TC-SF-NPs were obtained by using a powder X-ray diffractometer (Rigaku Ultima III, Rigaku Corporation, Tokyo, Japan). The measurements were recorded at a continuous scanning rate of 2°/min in the 2θ range of 5–45° (Jain et al., 2011).

#### 2.4.6. Stability of TC-SF-NPs

The stability studies of optimized TC-SF-NPs formulations were performed as per ICH guidelines. All the samples were stored in amber-colored vials at separate storage conditions. The study was performed at room temperature (25 ± 1 °C), elevated temperature (40 ± 3 °C) with 60 ± 3% relative humidity (RH), and at a lower temperature of (0–8 °C). Determination of particle size and zeta potential was also carried out at various time points to support any difference in mean diameter and zeta potential of NPs for 3 months.

#### 2.4.7. Lyophilization studies of TC-SF-NPs

The optimized formulation was freeze-dried without using any cryoprotectant. Freeze-dried TC-SF-NPs were collected and re-dispersed in deionized H_2_O. Particle size and zeta potential were obtained and S_f_/S_i_ (ratio of NP size after and before the freeze-drying process) was measured.

### 2.5. *In vitro* release studies

The *in vitro* release study of TC from SF-NPs was conducted in phosphate buffer solution of pH 5 and pH 7.4, as release media, to simulate the acidic environment within tumor tissue and physiological pH, respectively. In this experiment, 20 mg of free TC or lyophilized TC-loaded SF-NPs were dispersed in phosphate buffer saline, and the solution was placed in a dialysis membrane (MWCO 12,000–14,000 Da). The membrane was placed in the release medium under stirring at 50 rpm for 48 h. The temperature of the release media was maintained at 37 ± 0.5 °C. At pre-determined intervals (1, 2, 3, 4, 6, 12, 24, and 48 h), aliquots were collected from the release media and replaced with the same quantity of fresh PBS to maintain sink condition. The drug content in each sample was quantified using HPLC system.

### 2.6. *In vitro* cytotoxicity study

A non-cancerous epithelial cell line (MCF-10A) and cancerous MCF-7 and MDA-MB-231 human breast cancer cell lines were provided from the National Center for Cell Sciences (NCCS) (Pune, India). The cells were cultured in Dulbecco’s modified Eagle’s (DMEM) medium supplemented with 10% fetal bovine serum, 1% penicillin, and streptomycin at 37 °C. The *in vitro* cytotoxicity studies were evaluated using MTT assay (Abu Lila et al., 2021). Briefly, MCF-10A, MCF-7 and MDA-MB-231 cells (2 × 10^3^ cells/well) were seeded onto 96-well plate for 24 h. After which, the culture medium was removed and was replaced with fresh medium containing serial concentrations (25–400 μg/ml) of pure TC, blank SF-NPs or TC-SF-NPs, and the cells were further incubated at 37 °C in a 5% of CO_2_ incubator. At 72 h post-incubation, the medium was removed and 50 μl of MTT reagent (0.5 mg/ml) were added to each well and the cells were incubated at 37 °C for 4 h. Then, 150 μl of DMSO solution was added to each well to dissolve formazan crystals. The absorbance of each well was read at 570 nm using ELISA reader (ELX-800 Biotek, Winooski, VT). The data are presented as mean ± SD calculated from tree samples per condition.

### 2.7. Cell cycle analysis

MCF-7 and MDA-MB-231 (2 × 10^5^ cells/well) were cultured in a six-well plate and incubated at 37 °C in a 5% of CO_2_ incubator. At 24 h post incubation, the culture medium was replaced with fresh medium containing either free TC or TC-SF-NPs at IC_50_ concentrations, and the cells were further incubated for 24 h. After specified incubation time, cells were collected and centrifuged. The supernatant was discarded and cell pellets were rinsed twice with cold phosphate-buffered saline (PBS). Cells were re-suspended in 200 µl PBS and were subsequently fixed in 70% pre-cooled ethyl alcohol for 2 h at −20 °C. Cells were centrifuged for 5 min at 1000 rpm, the supernatant was disposed off, and the cells were rinsed trice with PBS. The obtained cell pellets were further incubated with a mixture of 400 μl PI/RNase (320 µl PI and 80 µl ribonuclease A) for 30 min in dark. The stained cells were analyzed by BD FACSCalibur^TM^ flow cytometer (BD Biosciences, San Jose, CA) and data was analyzed using CytExpert software. Data were presented by percentage of cells compared to the populations of the untreated cells.

### 2.8. *In vitro* cellular uptake studies

MCF-7 and MDA-MB-231 (2 × 10^5^ cells/well) were cultured in a six-well plate and incubated at 37 °C in a 5% of CO_2_ incubator. At 24 h post incubation, the culture medium was replaced with fresh medium containing 25 μg/ml PI-labeled TC-SF-NPs, and the cells were further incubated for 24 h. After specified incubation time, cells were collected and centrifuged. The supernatant was decanted and cells were rinsed twice with PBS. Untreated cells were used as control. Cellular uptake of PI-labeled TC-SF-NPs was analyzed using BD FACSCalibur^TM^ flow cytometer.

To visualize the intracellular uptake of TC-SF-NPs by breast cancer cells, confocal microscopy was utilized. Briefly, MCF-7 and MDA-MB-231 cells (10,000 cells per well) were seeded in 6-well glass slide (LabTek II, Farmingdale, NY) , incubated at 37 °C for 24 h. The cells were then treated with PI-labeled TC-SF-NPs (25 μg/ml) and further incubated for 24 or 48 h. After specified incubation time, the medium was decanted and the cells were washed twice with PBS. The cells were then fixed with 4% cold paraformaldehyde for 15 min at room temperature. Fixed cells were covered by cover slips and cellular uptake of TC-SF-NPs was inspected using an Advanced Spectral Confocal Microscope (Zeiss LSM 710, Zeiss, Oberkochen, Germany) (Abu Lila et al., 2021).

### 2.9. Cell apoptosis analysis by flow cytometry

MCF-7 and MDA-MB-231 breast cancer cells (3 × 10^5^ cells/well) were seeded in a six-well plate and were incubated at 37 °C in a 5% CO_2_ atmosphere. At 24 h post incubation, cultured cells were treated with either free TC or TC-SF-NPS (25 μg/ml) and further incubated for 24 h. The cells were harvested by trypsinization and rinsed trice with PBS. Subsequently, cells were stained by 5 µl Annexin V-FITC for 10 min and 10 µl PI for 5 min, respectively, in the dark at room temperature, according to manufacturer’s protocol (ThermoFisher Scientific, Waltham, MA). Analysis of cell apoptosis was carried out using flow cytometry (BD FACSCalibur^TM^; BD Biosciences, San Jose, CA). Data collection and analysis is done with CellQuest Pro software version 6.0 (BD Biosciences, San Jose, CA). The results are expressed as the rate of apoptosis (the percentage of early + late apoptotic cells) (Al Saqr et al., 2021a,b).

### 2.10. Statistical analysis

The data are presented as the mean ± SD. Statistical analysis was conducted by Student’s t-test or ANOVA. *p* < .05 was considered significantly different.

## 3. Result and discussion

### 3.1. Characterization of TC-SF-NPs

#### 3.1.1. Particle size and zeta potential

Blank SF-NPs and TC-loaded SF-NPs were characterized by dynamic light scattering (DLS) to estimate their hydrodynamic diameter, polydispersity index, and zeta potential. As shown in Table 1, the mean particle size of drug-loaded SF-NPs was remarkably higher than that of drug-free (blank) SF-NPs, which might be ascribed to the entrapment of drug molecules within NPs. The average particle size of TC-SF-NPs was 186.1 ± 5.9 nm, compared to 172.2 ± 4.3 of blank SF-NPs. In addition, the PDI values of SF-NPs and TC-loaded SF-NPs were 0.092 ± 0.02 and 0.169 ± 0.01, respectively, indicating homogenous size distribution (Abdallah et al., 2021).

**Table 1.** Physical characterization of blank SF-NPs, TC-SF-NPs, and lyophilized TC-SF-NPs.

Sample	Particle size (nm)	Polydispersity index	Zeta potential (mV)
Blank SF-NPs	172.2 ± 4.3	0.092 ± 0.02	−17.4 ± 1.1
TC-SF-NPs	186.1 ± 5.9	0.169 ± 0.01	−19.9 ± 1.4
Lyophilized TC-SF-NPs	195.1 ± 5.7	0.163 ± 0.01	−20.8 ± 1.9

Data represent mean ± SD of three independent experiments.

Zeta potential is a key indicator of the stability of colloidal dispersions. Generally, a zeta value of ±15 mV is needed for colloidal stability of nanoparticles (Soliman et al., 2020). The zeta potential of both blank SF-NPs and drug-loaded SF-NPs was in the same range and highly negative. The zeta potentials of blank SF-NPs and TC-SF-NPs were −17.4 ± 1.1 and −19.9 ± 1.4 mV, respectively, indicating high colloidal stability (Xiao et al., 2016).

For better storage stability, prepared drug-loaded SF-NPs were freeze-dried and were stored as a lyophilized powder ready for reconstitution. Accordingly, the effect of lyophilization on the average particle size and zeta potential of the lyophilized NPs was estimated. As shown in Table 1, there was a slight increase in both the particle size and zeta potential of lyophilized NPs, compared to freshly prepared ones. The ratio of NP size after and before the freeze-drying process (S_f_/S_i_) was 1.048, indicating the absence of particles agglomeration upon storage.

#### 3.1.2. Surface morphology of TC-SF-NPs

**Figure 2.**
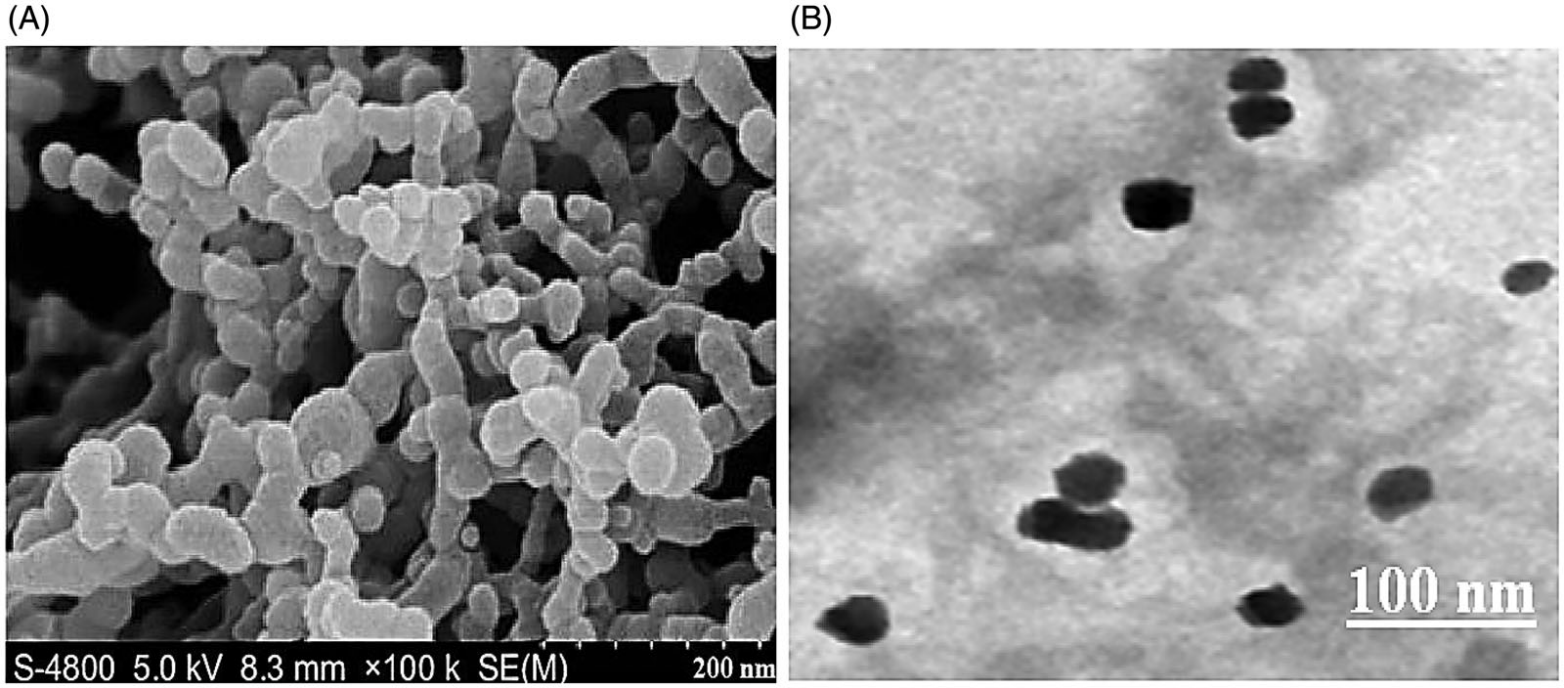
Surface morphology of TC-SF-NPs. (A) SEM image of TC-SF-NPs at X 100,000 (B) TEM of TC-SF-NPs. TC-SF-NPs: tamoxifen citrate silk fibroin nanoparticles; SEM: scanning electron microscopy; TEM: transmission electron microscopy.

Surface morphology of the prepared TC-SF-NPs was visualized using both SEM and high-resolution TEM. SEM images of TC-SF-NPs show that nanoparticles are spherical with a smooth surface (Figure 2(A)). Similarly, TC-SF-NPs were round shaped under TEM with a dense core and a thin corona (Figure 2(B)). Of interest, SEM and TEM images revealed smaller size of TC-SF-NPs compared with DLS results. DLS determines the hydrodynamic diameter of the particle core and the solvent layer attached to the particle, whereas SEM and/or TEM estimates the size of the dried sample without any hydration layer. Accordingly, the size obtained by SEM/TEM will always be smaller than the size estimated by DLS (Soliman et al., 2020).

#### 3.1.3. Fourier-transform infrared spectroscopy (FTIR)

**Figure 3.**
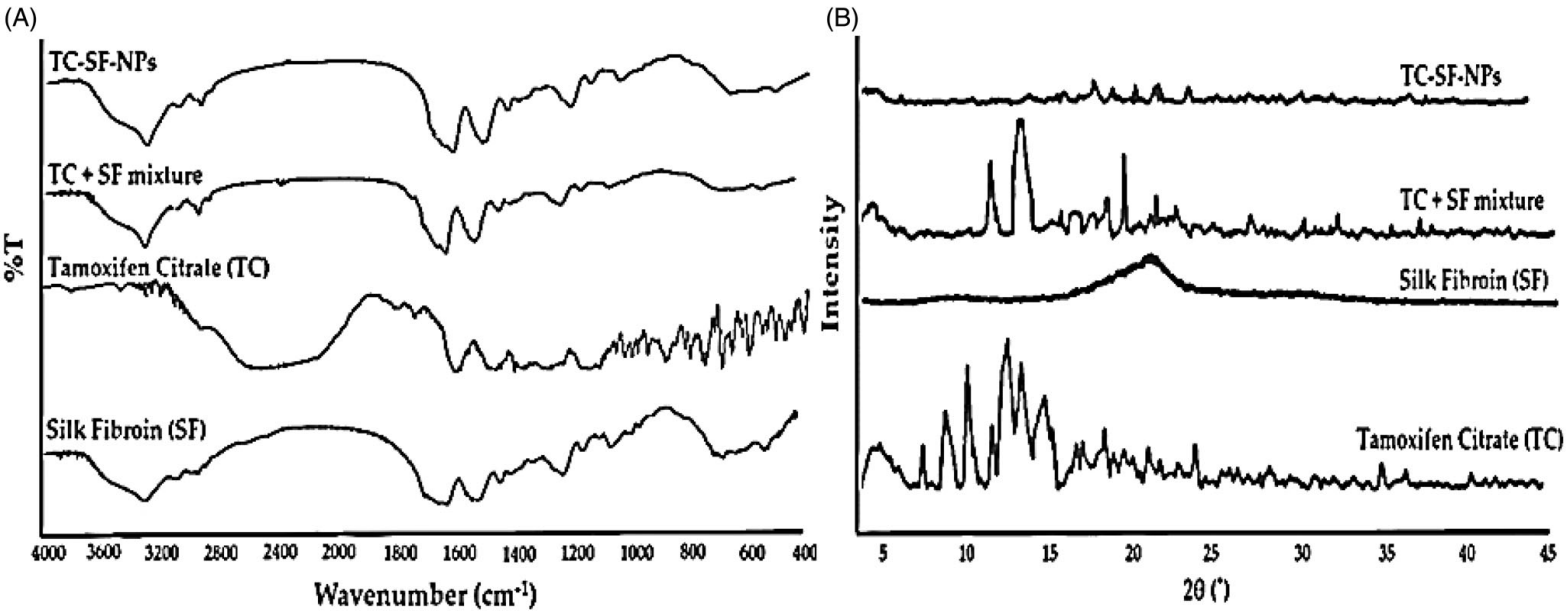
Physicochemical characterization of TC-SF-NPs. (A) Fourier transform infrared spectra. (B) X-ray diffraction spectra. TC-SF-NPs: tamoxifen citrate silk fibroin nanoparticles.

FTIR spectroscopy was adopted to confirm whether nanoparticles retain β–sheet structure of SF after the loading of TC. FTIR spectra of pure TC, SF, physical mixture of TC + SF and TC-loaded SF-NPs were presented in Figure 3(A). The FTIR spectra of SF exhibits typical peaks of crystalline β-sheet at 1539 cm^−1^ (amide II), 1623 cm^−1^ (amide-I), and 1231 cm^−1^ (amide-III), demonstrating that SF was in its stable conformation (Al Saqr et al., 2021a,b). FTIR spectrum of TC shows characteristic absorption peaks at 3094 cm^−1^ (N–H stretching), at 1723 cm^−1^ (C=O stretching), at 1594 cm^−1^ (N– bend), and at 1050 cm^−1^ (C–N stretch). Of interest, the FTIR spectrum of drug-loaded SF-NPs retained the characteristic absorption peaks of SF *viz* absorption peaks at 1633 and 1547 cm^−1^ corresponding to amide I and amide II, respectively, and the spectrum was similar to that of the physical mixture of TC + SF. These results indicate that the β–sheet in SF-NPs was not modified upon TC loading (Montalbán et al., 2018).

#### 3.1.4. X-ray diffraction analysis

XRD analysis was carried out to ascertain the possible changes in drug crystallinity upon loading onto SF-NPs. The X-ray diffractograms of pure drug, SF, physical mixture of TC and SF, and TC-SF-NPs are represented in Figure 3(B). The X-ray diffractogram of pure TC showed sharp crystalline peaks, confirming the crystalline nature of the drug. Of interest, the disappearance of characteristic crystalline peaks of TC in TC-SF-NPs indicated that the drug was encapsulated in SF-NPs in an amorphous state (Wu et al., 2018).

#### 3.1.5. Drug loading and entrapment efficiency

Drug loading/entrapment is one of the value criteria for estimating the quality of the drug delivery system. SF-based nanoparticles have been reported as a promising drug carrier for various drugs, especially hydrophobic drugs (Hardy et al., 2008; Mathur & Gupta, 2010). In this study, the drug loading percentage and the entrapment efficiency of TC within SF-NPs were 38.29 ± 0.63% and 79.08 ± 1.2%, respectively. SF is a natural polymer with bulky repetitive hydrophobic domains, which are interrupted by small hydrophilic groups (Mathur & Gupta, 2010; Zhao et al., 2015). Consequently, the relatively high entrapment efficiency of TC within SF-NPs might be ascribed to the hydrophobic nature of TC, which might favor its attachment to the bulk hydrophobic domains of SF *via* hydrophobic interactions and π–π stacking (Wenk et al., 2011). Of note, the entrapment efficiency of freeze-dried TC-SF-NPs was comparable to that of freshly prepared TC-SF-NPs; 78.82 ± 0.8% *vs.* 79.08 ± 1.2%, respectively.

### 3.2. In vitro release studies

**Figure 4.**
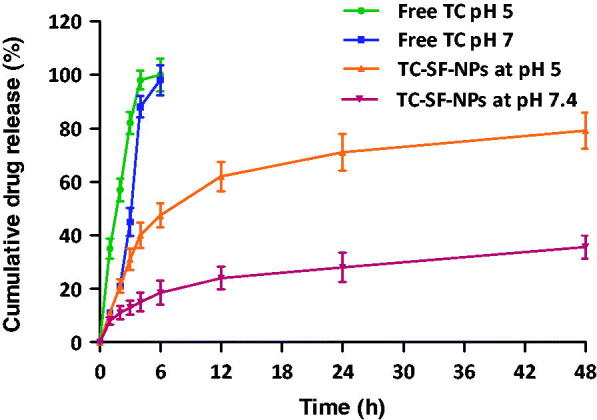
*In vitro* release study of free TC and TC-loaded SF-NPs at different pH. TC: tamoxifen citrate; TC-SF-NPs: tamoxifen citrate silk fibroin nanoparticles.

Controlled release drug delivery systems are evidently designed to ensure the release of the entrapped drug in specified amounts over a defined period of time. Such systems bear the advantages of sustaining drug release to maintain adequate concentrations of the drug at the target site for a prolonged period of time, which is of considerable significance for the treatment of chronic disorders. In addition, sustaining drug release could minimize the administration frequency as well as adverse drug effects, leading to improved patient compliance (Yucel et al., 2014). In this study, *in vitro* release pattern of TC from TC-loaded SF-NPs was examined at two different pH values, pH 7.4 and pH 5, in order to mimic the physiological pH and tumor-associated pH environment, respectively. As shown in Figure 4, a burst drug release from SF-NPs was observed at both pH values with 18.56 ± 1.6% and 47.48 ± 4.5% of the entrapped drug were release during the first 6 h at pH 7.4 and pH 5, respectively. The initial burst drug release from SF-NPs might be attributed, on the one hand, to rapid release of drug molecules that are located at the hydrophobic–hydrophilic interface of SF-NPs, and on the other hand, to the higher drug entrapment efficiency, which might trigger faster drug release. Nevertheless, SF-NPs could efficiently sustain drug release for up to 48 h, compared to free drug, where, 35.62 ± 6.2% and 79.15 ± 8.7% of entrapped drug were released at the end of 48 h release time at pH 7.4 and pH 5, respectively. In addition, silk nanoparticles have been reported to show pH-dependent release *in vitro* (Seib et al., 2013). As shown in Figure 4, TC release was significantly faster from SF-NPs when incubated in an acidic medium (pH 5.0) than at pH 7.4; up to 60% of the loaded drug were released in the first 12 h at pH 5.0, whilst, less than ≈20% of the entrapped drug was released from SF-NPs at pH 7.4 during the same time period. At low pH values, SF loses their overall acidic surface properties and has negative net charges (Seib et al., 2012), which in turn compromises the electrostatic interactions between TC and SF, resulting in a significant increase in drug release. Consequently, SF nanoparticles could elicit a differential drug release in response to pH changes without the need to be chemically modified.

### 3.3. Stability of prepared TC-SF-NPs

One of the most significant considerations in the manufacture of pharmaceutical formulations is the stability of polymeric materials. While, due to their biocompatibility and biodegradability, natural polymers are preferred to their synthetic alternatives for clinical applications, they must still follow stability standards to be eligible for use in the pharmaceutical industry. Consequently, stability studies for TC-SF-NPs were carried out for 3 months according to ICH guidelines and the obtained data are summarized in Table 2. Slight increase in particle size and zeta potential of the prepared TC-SF-NPs was observed at the end of three months at all storage conditions. In addition, there were only 3% difference in the percentage drug release after exposing the NPs at 40 ± 2 °C and 75 ± 5% RH. Furthermore, there was negligible change in drug content at accelerated temperature when compared to the long term or refrigeration. From the results, it was confirmed that TC-SF-NPs are fairly stable at different storage conditions.

**Table 2.** Stability study of optimized TC-SF-NPs at different temperatures and the humidity condition.

Stability condition	Time (month)	Particle size (nm)	Zeta potential (mV)	Initial amount of drug (mg)	Drug amount at the end of 3 months (mg)	% Degraded at the end of 3 months
25 ± 2 °C/60 ± 5% RH	0	186.12 ± 5.87	−19.21 ± 0.8	7.51 ± 0.12	7.42 ± 0.19	1.20
	1	187.31 ± 5.64	−19.42 ± 0.7			
	3	189.24 ± 4.80	−20.02 ± 1.1			
40 ± 2 °C/ 75 ± 5% RH	0	186.12 ± 5.87	−19.21 ± 0.6	7.51 ± 0.12	7.36 ± 0.13	1.99
	1	187.53 ± 6.25	−19.42 ± 0.9			
	3	191.14 ± 4.86	−20.36 ± 1.3			
5 ± 2 °C/ambient RH	0	186.12 ± 5.87	−19.21 ± 1.2	7.51 ± 0.12	7.46 ± 0.16	0.66
	1	187.63 ± 5.46	−19.42 ± 0.8			
	3	187.54 ± 4.39	−20.16 ± 1.0			

Data are given in mean ± SD (*n* = 3).

### 3.4. *In vitro* cytotoxicity

**Figure 5.**
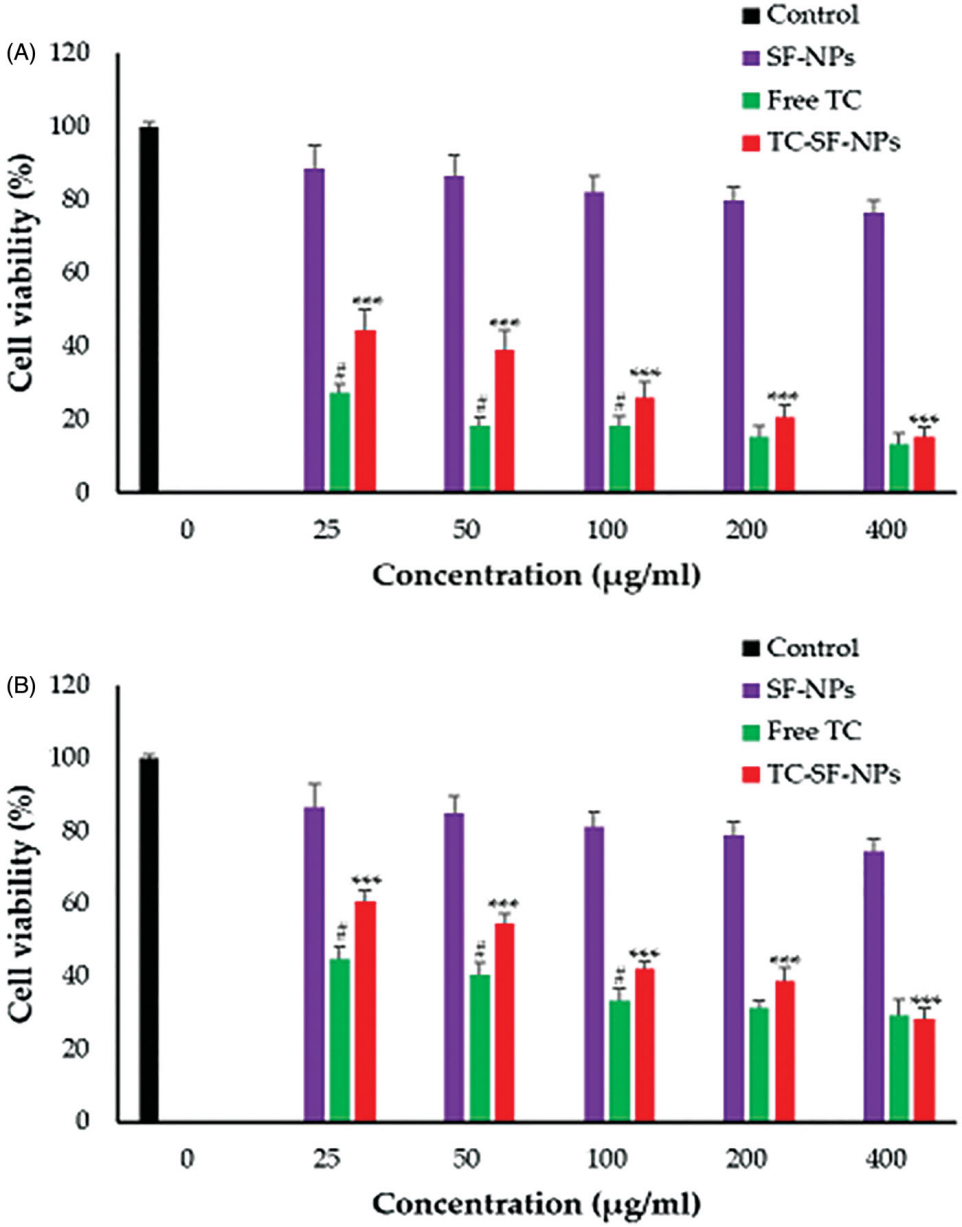
*In vitro* cytotoxicity of TC-SF-NPs against breast cancer cell lines. (A) MCF-7 and (B) MDAMB-231 cells were incubated with different concentrations (25–400 μg/ml) of free TC, SF-NPs or TC-loaded SF-NPs for 72 h. Untreated cells served as controls. The dose of TC in TC-SF-NPs group was equivalent to that of free TC group. Cell viability was assessed by MTT assay. Data represent mean ± SD. ^#^*p* < .05 *vs.* TC-SF-NPs, ***p < .005 *vs.* SF-NPs. TC: tamoxifen citrate; SF-NPs: silk fibroin nanoparticles; TC-SF-NPs: tamoxifen citrate silk fibroin nanoparticles.

In this study, non-cancerous MCF-10A epithelial cells and cancerous MCF-7 and MDA-MB-231 breast cancer cell lines were exposed to free TC, drug-free SF-NPs and TC-loaded SF-NPs at various concentrations (25–400 μg/ml) for 72 h. The cell viability was then tracked by MTT assay. The relative cell viability was calculated by assuming the control sample had 100% viable cells. As shown in Figure 5, drug-free SF-NPs did not exert any obvious cytotoxicity against all the tested cell lines, suggesting the safety of SF-NPs. On the other hand, free TC and TC-loaded SF-NPs exerted a potent concentration-dependent cytotoxic effect against both MCF-7 and MDA-MB-231 cancer cell lines. TC-SF-NPs at high concentrations (400 μg/ml) showed a remarkable decrease in cell viability of both cell lines (<80%). The IC_50_ values of TC against MCF-7 and MDA-MB-231 cells were 56.49 and 86.83 µg/ml, respectively, while, the IC_50_ values of TC-loaded SF-NPs against MCF-7 and MDA-MB-231 cells were 86.91  and 107.17 µg/ml, respectively. The relatively higher IC_50_ values of TC-SF-NPs, compared to free TC (exhibiting a zeta potential of −8.3 ± 1.2 mV), might be attributed, on the one hand, to the lower cellular interactions between the negatively charged SF-NPs and cancer cells, and, on the other hand, to the sustained release of relatively lower TC concentrations from SF-NPs over a longer period of time. Of interest, TC-SF-NPs did not exert remarkable cytotoxicity against noncancerous MCF-10A cells; cell viability was >85% even upon treatment with 400 μg/ml for 72 h (Supplementary Figure 1). Collectively, our results emphasized the potent cytotoxic effect of TC-SF-NPs against breast cancer cells.

### 3.5. *In vitro* cellular uptake studies of nanoparticles

**Figure 6.**
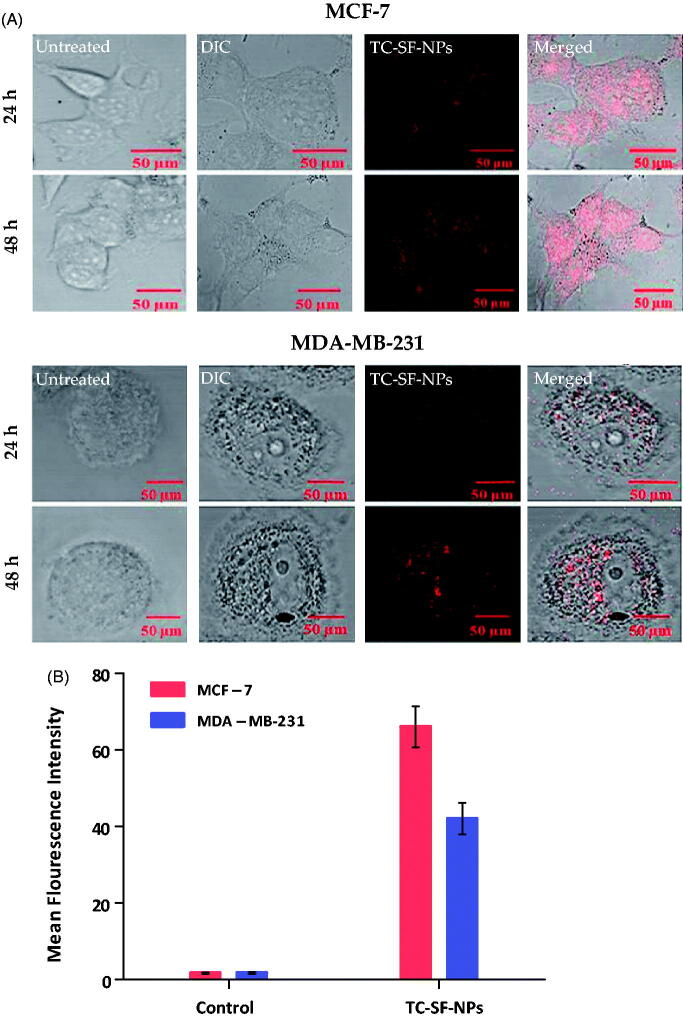
Cellular uptake of TC-SF-NPs by MCF-7 and MDAMB-231 cell lines. (A) Confocal microscopy images of MCF-7 and MDA-MB-231 cells treated with PI-labeled TC-SF-NPs for 24 and 48 h. (B) Flow cytometry quantitative analysis showing percentage of uptake of PI-labeled TC-SF-NPs by MCF-7 and MDA-MB-231 cells following 24 h incubation. Data are expressed in mean ± SD. **p < .01. TC: tamoxifen citrate; TC-SF-NPs: tamoxifen citrate silk fibroin nanoparticles.

Cell internalization/uptake of drug *via* nanoparticles is a well-recognized strategy to enhance the therapeutic potential of cytotoxic agents. In this study, MCF-7 and MDA-MB-231 cells were incubated with 25 μg/ml of PI-labeled TC-SF-NPs for 24 and 48 h, and the cellular uptake of SF-NPs was probed qualitatively and quantitatively by confocal microscopy and flow cytometry. Figure 6(A) represents confocal microscopy images of the internalization of TC-loaded SF-NPs. In the control cells, no red fluorescence signals were observed. On the other hand, intense red fluorescence was detected in both cancer cell lines incubated with TC-SF-NPs, indicating efficient uptake/internalization of SF-NPs by both cell lines. In addition, higher fluorescent signals were observed in both cell lines upon increasing the duration of incubation up to 48 h; with higher fluorescence intensity observed in MCF-7, compared to MDA-MB-231 cells, signifying better uptake of TC-SF-NPs by MCF-7 cells. Of interest, PI-related fluorescence signals indicated preferential localization of TC-SF-NPs in the cytoplasm and perinuclear region of both cell lines.

Furthermore, flow cytometry analysis was adopted to quantitatively evaluate the cellular uptake of PI-labeled TC-SF-NPs by MCF-7 and MDA-MB-231 breast cancer cells. The cellular uptake of PI-labeled TC-SF-NPs was ∼66% and ∼42% in MCF-7 and MDA-MB-231 cells, respectively, after incubated them for 24 h (Figure 6(B)). Collectively, our results suggest that the superior cytotoxic effect of TC-loaded SF-NPs against MCF-7 cells, compared to MDA-MB-231 cells, might be ascribed to the preferential cellular uptake of SF-NPs by MCF-7 cells.

### 3.6. Cell cycle analysis

Flow cytometry analysis was adopted to evaluate the effect of TC-loaded SF-NPs on the cell cycle progression of MCF-7 and MDA-MB-231 breast cancer cells. Table 3 summarizes the relative percentages of MCF-7 and MDA-MB-231 cells in each phase of the cell cycle following treatment. Consistent with previous reports (Osborne et al., 1983), treatment with free TC for 24 h effectively induced cells accumulate in G1 phase, with a concomitant depletion of S- and G2/M-phase in both MCF-7 and MDA-MB-231 breast cancer cells, compared to control (untreated) cells. Of interest, TC-SF-NPs induced a significant increase in the percentage of cells in the G_0_/G_1_ phase, which was superior to that of free TC. The mean percentage of cells in the G_0_/G_1_ phase of the cell cycle were 45.30 ± 2.07 and 44.70 ± 2.95 for MCF-7 and MDA-MB231 cells, respectively, upon treatment with TC-loaded SF-NPs, compared to 41.18 ± 1.71 and 39.25 ± 1.98 for cells treated with free TC, respectively. In addition, G_0_/G_1_ phase cell cycle arrest was accompanied with a mutual reduction in the percentage of cells in the S and G_2_/M phases of the cell cycle upon treatment with TC-SF-NPs. These results suggest that, following treatment with TC-SF-NPs, breast cancer cells were encumbered in their cell cycle progression and were accumulated in the G_0_/G_1_ phase. Similar results were represented by Osborne et al., who reported that tamoxifen inhibits MCF-7 cell proliferation by invoking a transition delay or block in the early-to-mid-G_1_ phase of the cell cycle (Osborne et al., 1983).

**Table 3.** Effect of free TC and TC-loaded SF-NPs on the redistribution of growth-arrested MCF-7 and MDA-MB-231 cells in the different phases of the cell cycle.

Cell cycle phase	MCF-7 Cells			MDA-MB-231 Cells		
	Control	Free TC	TC-SF-NPs	Control	Free TC	TC-SF-NPs
Sub G_0_/G_1_	11.48 ± 0.77	14.32 ± 0.81**	18.11 ± 0.94^**, #^	10.72 ± 0.48	14.12 ± 1.08**	18.58 ± 1.03^**, #^
G_0_/G_1_	36.10 ± 1.25	41.18 ± 1.71**	45.30 ± 2.07^**, #^	34.88 ± 2.23	39.25 ± 1.98**	44.70 ± 2.95^**, #^
S	25.31 ± 1.13	20.92 ± 1.23**	16.28 ± 0.65^**, #^	24.45 ± 1.76	19.75 ± 0.98**	16.95 ± 1.13^**, #^
G_2_/M	27.11 ± 1.45	23.58 ± 2.01**	20.31 ± 1.98^**, #^	29.95 ± 2.56	26.88 ± 1.34**	19.77 ± 0.85^**, #^

Data represent percentage of single-cell events (mean ± SD) in the four different phases. **p < .01 *vs.* control, *^#^p* < .05 *vs.* free TC.

### 3.7. Cell apoptosis study

**Figure 7.**
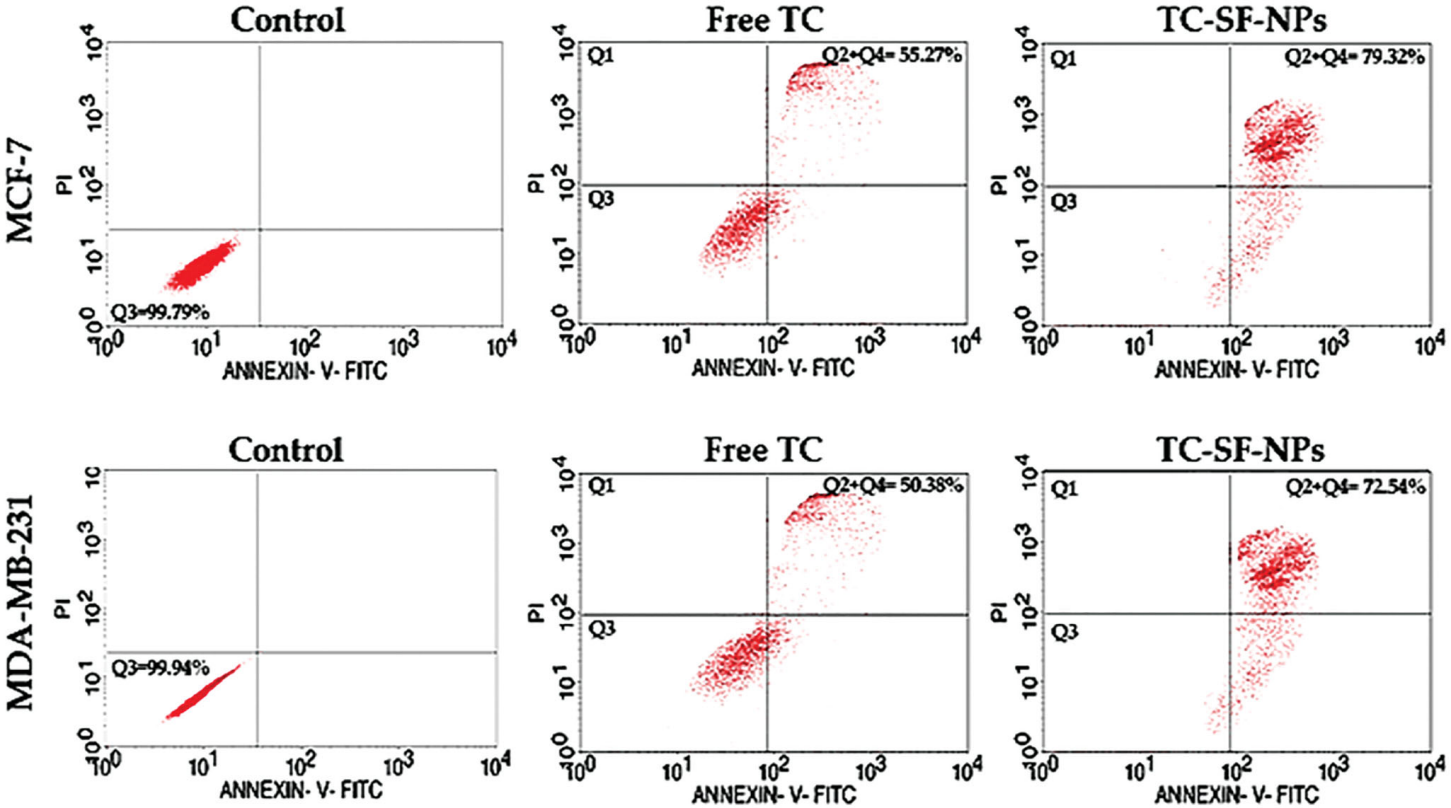
Cellular apoptosis of breast cancer cells treated with TC-loaded SF-NPs. MCF-7 and MDA-MB-231 cells (3 × 10^5^ cells/well) were treated with either free TC or TC-SF-NPS for 24 h. Cellular apoptosis was evaluated by flow cytometry using Annexin V-FITC staining. TC: tamoxifen citrate; TC-SF-NPs: tamoxifen citrate silk fibroin nanoparticles.

In order to validate the efficacy of drug-loaded nanoparticles in the treatment of cancer, their effectiveness was tested at the cellular level by evaluating cell susceptibility to cell apoptosis. In this study, MCF-7 and MDA-MB-231 cells were exposed to free TC or TC-loaded SF-NPs for 24 h and cellular apoptosis was assessed by flow cytometry analysis using Annexin V-FITC/PI double staining. As shown in Figure 7, both free TC and TC-loaded onto SF-NPs were capable of inducing apoptosis in both cell lines. In addition, TC-SF-NPs induced more apoptotic response against both cell lines compared to free TC. The rate of apoptosis induced by TC-loaded SF-NPs was 79.32% and 72.54%, respectively, against MCF-7 and MDA-MB-231 cells, compared to 55.27% and 50.35% induced by free TC after 24 h-incubation. These results suggest that loading of TC onto SF-NPs did not compromise the cytotoxic activity of TC against cancer cell lines. Of interest, flow cytometry analysis results confirmed that initiation of apoptosis was related to variation in the cell cycle progression. Accumulating evidences indicated that alteration in the cell cycle might prevent or induce apoptosis and/or necrosis (Vermeulen et al., 2003; Yang et al., 2019). In this study, it was evident that cell cycle arrest at G_0_/G_1_ phase resulted in cell apoptosis rather than cell necrosis.

## 4. Conclusion

In this study, TC-SF-NPs were prepared, characterized, and evaluated for their effectiveness against breast cancer cell lines. The prepared TC-SF-NPs were fairly stable at different storage conditions. *In vitro* release studies verified a biphasic release pattern with an initial burst effect followed by a sustained release for 48 h. The biosynthesized TC-loaded SF-NPs showed cytotoxic efficacy against breast cancer cell lines. In addition, cell cycle analysis confirmed that the anti-tumor activity of TC-SF-NPs is mediated mainly *via* arresting the G0/G1 phase with subsequent induction of cellular apoptosis. Collectively, TC-SF-NPs are expected to efficiently restrain tumor progression at a lower dose than required for conventional oral tamoxifen treatment, and consequently, could minimize treatment-related side effects. Nevertheless, further studies are urgently needed to support our observations of the anti-tumor potential of silk fibroins nanoparticles *in vivo*.

## Supplementary Material

Supplemental Material
