# Supplementary material for: Formulation, characterization, and cellular toxicity assessment of tamoxifen-loaded silk fibroin nanoparticles in breast cancer
Source: Drug Deliv. 2021 Jul 30;28(1):1626–36. doi: 10.1080/10717544.2021.1958106 (PMC8330732; doi:10.1080/10717544.2021.1958106)
Supplement: Supplemental Material [file IDRD_A_1958106_SM3443.docx]

**Supplementary Figure 1.** *In vitro* cytotoxicity of SF-NPs and TC-loaded SF-NPs against non-cancerous MCF-10A epithelial cells. MCF-10A cells were incubated with different concentrations (25-400 μg/ml) of SF-NPs or TC-loaded SF-NPs for 72 h. Cell viability was assessed by MTT assay. Data represent mean ± SD.
